# SERPINA12 in skin: molecular mechanisms and roles in adipocytes, psoriasis, and palmoplantar keratoderma

**DOI:** 10.3389/fimmu.2026.1812302

**Published:** 2026-04-27

**Authors:** Zhenzhen Xiao, Fei Wang, Rui Li, Yingjian Tan

**Affiliations:** 1 Department of Dermatology, Fuzhou First General Hospital, Fuzhou, China; 2 Beijing Pinsu Medical Aesthetic Clinic, Beijing, China; 3 Department of Dermatology, Venereology and Allergology, Charité-Universitätsmedizin Berlin, Berlin, Germany

**Keywords:** gene therapy, KLK7, palmoplantar keratoderma, psoriasis, SERPINA12

## Abstract

SERPINA12 is a member of the serpin superfamily that has been extensively studied in metabolic and inflammatory disorders. In recent years, increasing evidence has highlighted its emerging role in skin physiology and dermatological diseases. SERPINA12 is expressed in multiple skin cell types, including keratinocytes and dermal fibroblasts, where it participates in the regulation of inflammation, cellular proliferation, differentiation, and tissue homeostasis. Dysregulation of SERPINA12 has been implicated in several skin disorders. In psoriasis, altered SERPINA12 expression is associated with chronic inflammation, immune dysregulation, and abnormal keratinocyte proliferation, suggesting a potential modulatory role in psoriatic pathogenesis. Furthermore, emerging studies suggest a possible involvement of SERPINA12 in palmoplantar keratoderma, where it may contribute to aberrant keratinization and epidermal barrier dysfunction. This review summarizes current knowledge on the expression patterns, biological functions, and molecular mechanisms of SERPINA12 in the skin, with a particular focus on adipocytes, psoriasis, and palmoplantar keratoderma. Understanding the role of SERPINA12 in cutaneous biology may provide new insights into disease pathogenesis and identify potential therapeutic targets for skin disorders.

## 1 Introduction

SERPINA12, also known as vaspin (visceral adipose tissue–derived serine protease inhibitor), is a member of the serpin superfamily and was initially identified as an adipokine highly expressed in adipose tissue in 2005 (1). Since its discovery, SERPINA12 has attracted increasing attention due to its pleiotropic roles across multiple organ systems. Early studies primarily focused on its involvement in metabolic regulation, demonstrating that SERPINA12 participates in the modulation of insulin sensitivity, glucose homeostasis, and lipid metabolism (2). Elevated circulating levels of SERPINA12 have been reported in obesity, type 2 diabetes, and metabolic syndrome, where it is generally considered a compensatory factor counteracting metabolic stress and inflammation (3–5).

**Figure 1:**
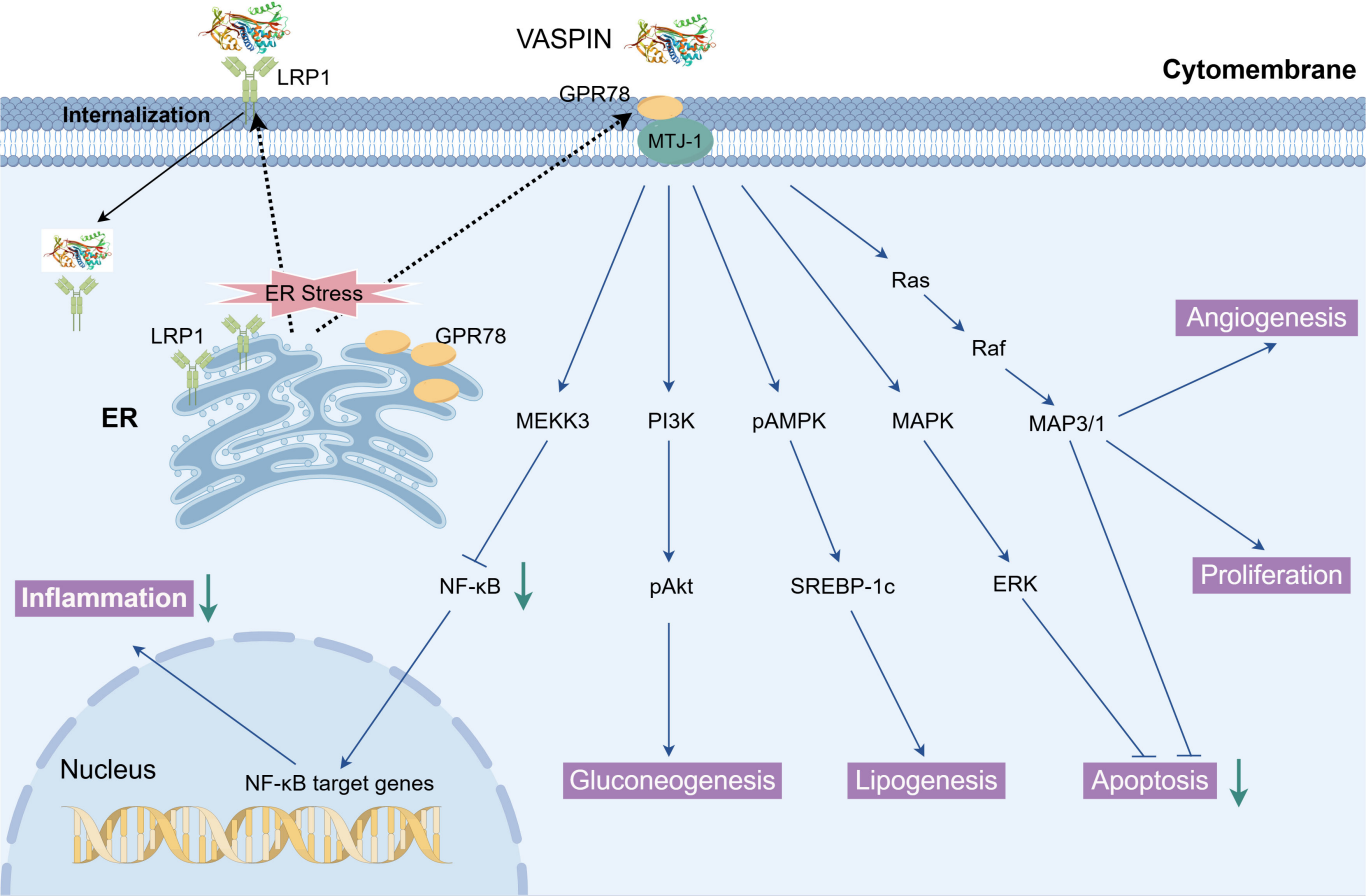
VASPIN-mediated signaling pathways and biological functions. VASPIN binds to cell-surface GRP78 (MTJ-1 complex) and LRP1, particularly under ER stress conditions, triggering receptor internalization and multiple downstream pathways. Activation of MEKK3/NF-κB suppresses inflammation, while PI3K/Akt and AMPK/SREBP-1c regulate gluconeogenesis and lipogenesis. The Ras/Raf/MAPK and ERK pathways further promote angiogenesis and proliferation and inhibit apoptosis, highlighting the diverse roles of VASPIN in metabolic and cellular regulation.

Beyond metabolic tissues, SERPINA12 has been implicated in a wide range of biological processes, including inflammation, oxidative stress, cell survival, and tissue remodeling. Mechanistically, SERPINA12 functions as a serine protease inhibitor, targeting specific proteases involved in inflammatory signaling and extracellular matrix regulation (6). In addition, SERPINA12 has been shown to modulate key intracellular pathways such as GRP78, NF-κB, STAT3, PI3K–AKT, and MAPK, thereby influencing cytokine production, cellular proliferation, and apoptosis(**Figure 1**). Through these mechanisms, SERPINA12 contributes to the maintenance of tissue homeostasis under both physiological and pathological conditions (7).

Emerging evidence further suggests that SERPINA12 acts as an endocrine and paracrine mediator, enabling crosstalk between adipose tissue and peripheral organs, including the liver, cardiovascular system, pancreas, and immune system (8–13). In these contexts, SERPINA12 exhibits anti-inflammatory and cytoprotective properties, attenuating excessive immune activation and limiting tissue damage. Dysregulation of SERPINA12 expression or function has been associated with chronic inflammatory diseases, fibrosis, and tumorigenesis, highlighting its broad relevance beyond metabolic disorders (7).

Given the central roles of inflammation, protease activity, and cellular differentiation in skin biology, it is not surprising that SERPINA12 has recently emerged as a molecule of interest in dermatological research. The skin is a highly dynamic organ that relies on tightly regulated protease–antiprotease balance, immune surveillance, and keratinocyte differentiation to maintain barrier integrity and immune defense (6). Disruption of these processes underlies the pathogenesis of many skin diseases, including hyperkeratotic disorders, inflammatory dermatoses, and skin cancers. Accumulating studies indicate that SERPINA12 is expressed in skin tissues since its first measurement in skin from 2012 (14) and may participate in the regulation of epidermal homeostasis, inflammatory responses, and the cutaneous microenvironment.

In particular, altered SERPINA12 expression has been linked to disorders characterized by abnormal keratinization and chronic inflammation, such as palmoplantar keratoderma and psoriasis, as well as to skin tumor development (7, 15, 16). These findings suggest that SERPINA12 may serve as a critical molecular link between systemic metabolic–inflammatory regulation and local skin pathology. A comprehensive understanding of SERPINA12 function in the skin may therefore provide novel insights into disease mechanisms and reveal potential therapeutic targets for a range of dermatological conditions.

## 2 Overview of SERPINA12 in skin

### 2.1 Evolutionary background of SERPINA12 in skin

The evolutionary trajectory of SERPINA12 (vaspin) in skin biology is intertwined with the emergence of cornified skin appendages—a defining innovation of terrestrial vertebrates. While SERPINA12 itself has not been directly linked to ancient tetrapod skin structures, comparative genomic studies of related serpin family members and skin differentiation regulators provide critical context. For example, the transcription factor Hoxc13, which controls the expression of hair keratins in mammals, was recently shown to regulate cornified claw formation in Xenopus frogs via conserved binding sites in keratin gene promoters (17). This suggests that the genetic networks governing cornified appendage development are deeply conserved across terrestrial vertebrates, potentially including serpins that modulate proteolytic balance in the epidermis.

In mammals, SERPINA12 belongs to the serpin superfamily, which includes protease inhibitors critical for skin barrier function. A key parallel is observed with PSORS1C2, a mammal-specific serpin located in the psoriasis susceptibility locus 1 (PSORS1). PSORS1C2 is expressed in the granular layer of the epidermis and is absent in aquatic mammals (e.g., whales and dolphins), where epidermal differentiation is degenerated (18). This pattern mirrors the tissue-specific expression of SERPINA12 in keratinocytes, suggesting that both serpins evolved to support the terrestrial vertebrate skin barrier by regulating proteolytic activity. While SERPINA12’s exact evolutionary origin remains to be fully elucidated, its role in inhibiting kallikrein-related peptidases (KLKs)—enzymes central to desquamation—aligns with the adaptive need to maintain epidermal integrity in dry, terrestrial environments.

### 2.2 Basic biological functions of SERPINA12 in skin

SERPINA12 (vaspin) is a serine protease inhibitor with multifaceted roles in skin homeostasis, primarily acting through the regulation of epidermal differentiation and inflammation (19). In healthy skin, vaspin expression is tightly linked to keratinocyte maturation: proliferating basal keratinocytes exhibit low vaspin levels, while differentiated suprabasal cells show high expression (16). This pattern is critical for maintaining the skin barrier, as vaspin inhibits KLK7 and KLK14—two proteases that degrade desmoglein-1 and corneodesmosin, key components of intercellular junctions in the epidermis (15, 20–22). Loss-of-function SERPINA12 variants disrupt this balance, leading to excessive KLK activity, reduced desmosomal proteins, and epidermal hyperkeratosis, as observed in autosomal recessive palmoplantar keratoderma (PPK) (15).

Beyond protease inhibition, vaspin exerts potent anti-inflammatory effects in the skin. In a mouse model of psoriasis-like inflammation, epidermal vaspin expression is significantly downregulated, and vaspin deficiency in keratinocytes upregulates interferon-inducible and pro-inflammatory genes (e.g., TNF-α, IL-1β, IL-6) (16). Conversely, exogenous vaspin application reduces myeloid cell infiltration and inflammatory cytokine secretion by dendritic cells, macrophages, and neutrophils (16). Mechanistically, vaspin suppresses the NF-κB pathway, as demonstrated in 3T3-L1 adipocytes where it inhibits IKKα/β phosphorylation and IκB degradation (23). This anti-inflammatory activity is further supported by clinical data: non-obese psoriasis patients have 99.72 pg/mg of tissue vaspin, compared to 257.34 pg/mg in healthy controls (p < 0.001), and NB-UVB treatment— which improves psoriasis—upregulates vaspin to 190.92 pg/mg (p < 0.001) (24).

### 2.3 Comparative analysis of SERPINA12 expression in skin tissues

SERPINA12 expression varies significantly across skin compartments and disease states, reflecting its context-dependent roles in homeostasis and pathology. In healthy human skin, vaspin is predominantly localized to the differentiated suprabasal layers of the epidermis, with minimal expression in the dermis (16, 24). This contrasts with SERPINE1 (another serpin), which is restricted to the basal layer in normal epidermis but upregulated in 96% of primary melanomas and 92% of metastatic melanomas (25). While direct comparative studies of SERPINA12 in different skin tumors are limited, its expression in hepatocellular carcinoma (HCC) CD133+ stem cells—where it drives AKT/β-catenin signaling—suggests potential tissue-specific oncogenic roles (26).

In inflammatory skin diseases, SERPINA12 expression is consistently downregulated in lesional vs. non-lesional skin. For example, in psoriasis vulgaris, tissue vaspin levels are 61.3% lower in lesional skin compared to healthy controls (99.72 pg/mg vs. 257.34 pg/mg, p < 0.001) (24). Similarly, in atopic dermatitis (AD), serum vaspin levels are reduced in adult patients, though the exact tissue expression pattern remains understudied (27). Cross-tissue comparisons also reveal species-specific differences: while mouse models of psoriasis-like inflammation show reduced epidermal vaspin (16), rats with diet-induced obesity exhibit increased vaspin expression in adipose tissue (23). These variations highlight the need for context-specific analyses of SERPINA12, as its function depends on both tissue localization and the presence of inflammatory or oncogenic stimuli.

## 3 Pathophysiological roles of SERPINA12 in skin disorders

### 3.1 SERPINA12 in adipocytes

SERPINA12 was originally identified as an adipokine predominantly expressed and secreted by adipocytes in 2005 (1). Adipocyte-derived SERPINA12 may influence skin biology through systemic metabolic regulation and inflammation, supporting the existence of a skin–adipose axis. It is highly enriched in visceral and subcutaneous adipose tissue and plays a crucial role in regulating adipocyte function, metabolic homeostasis, and inflammatory responses. In adipocytes, SERPINA12 expression is dynamically regulated by nutritional status, insulin sensitivity, and inflammatory cues, highlighting its importance in adipose tissue physiology (28).

One of the primary functions of SERPINA12 in adipocytes is the modulation of insulin sensitivity (1). Experimental studies have demonstrated that SERPINA12 improves insulin signaling by enhancing insulin receptor activity and downstream pathways, including the PI3K–AKT cascade. By inhibiting specific serine proteases involved in insulin resistance, SERPINA12 helps preserve insulin responsiveness in adipocytes, thereby promoting glucose uptake and lipid storage under physiological conditions. Consistently, increased SERPINA12 expression has been observed in states of obesity and insulin resistance, suggesting a compensatory mechanism aimed at counteracting metabolic stress (14).

In addition to its metabolic effects, SERPINA12 exerts significant anti-inflammatory functions within adipose tissue (29). Adipocytes are not only energy-storing cells but also active participants in immune and inflammatory processes. SERPINA12 has been shown to suppress the expression of pro-inflammatory cytokines such as TNF-α and IL-6 while attenuating the activation of inflammatory signaling pathways, including NF-κB and MAPK (14, 16, 23). Through these actions, SERPINA12 contributes to the maintenance of a balanced inflammatory microenvironment in adipose tissue, which is essential for normal adipocyte differentiation and function.

SERPINA12 also influences adipocyte differentiation and survival. During adipogenesis, its expression increases alongside key adipogenic transcription factors, suggesting a role in adipocyte maturation. Moreover, SERPINA12 has been reported to protect adipocytes from apoptosis induced by metabolic stress, oxidative damage, or inflammatory stimuli (30). This coordinated upregulation with transcription factors such as PPARγ and C/EBPα further implies that SERPINA12 may participate in the regulation of gene networks essential for lipid accumulation and the acquisition of mature adipocyte phenotypes (7, 28). In addition, SERPINA12 may contribute to maintaining cellular homeostasis during the differentiation process by modulating local inflammatory responses and protease activity within adipose tissue (12). This cytoprotective effect supports adipose tissue expansion in a controlled manner and prevents excessive adipocyte loss, which could otherwise exacerbate ectopic lipid deposition and metabolic dysfunction (31).

Beyond local effects, SERPINA12 functions as a circulating adipokine that mediates crosstalk between adipose tissue and other organs, including the liver, pancreas, vascular endothelium, and skin (30). By acting in an endocrine and paracrine fashion, SERPINA12 links adipocyte metabolism to systemic inflammatory and metabolic regulation. These properties underscore its relevance not only in metabolic disorders but also in inflammatory diseases affecting peripheral tissues.

### 3.2 SERPINA12 in inflammatory skin diseases

**Figure 2:**
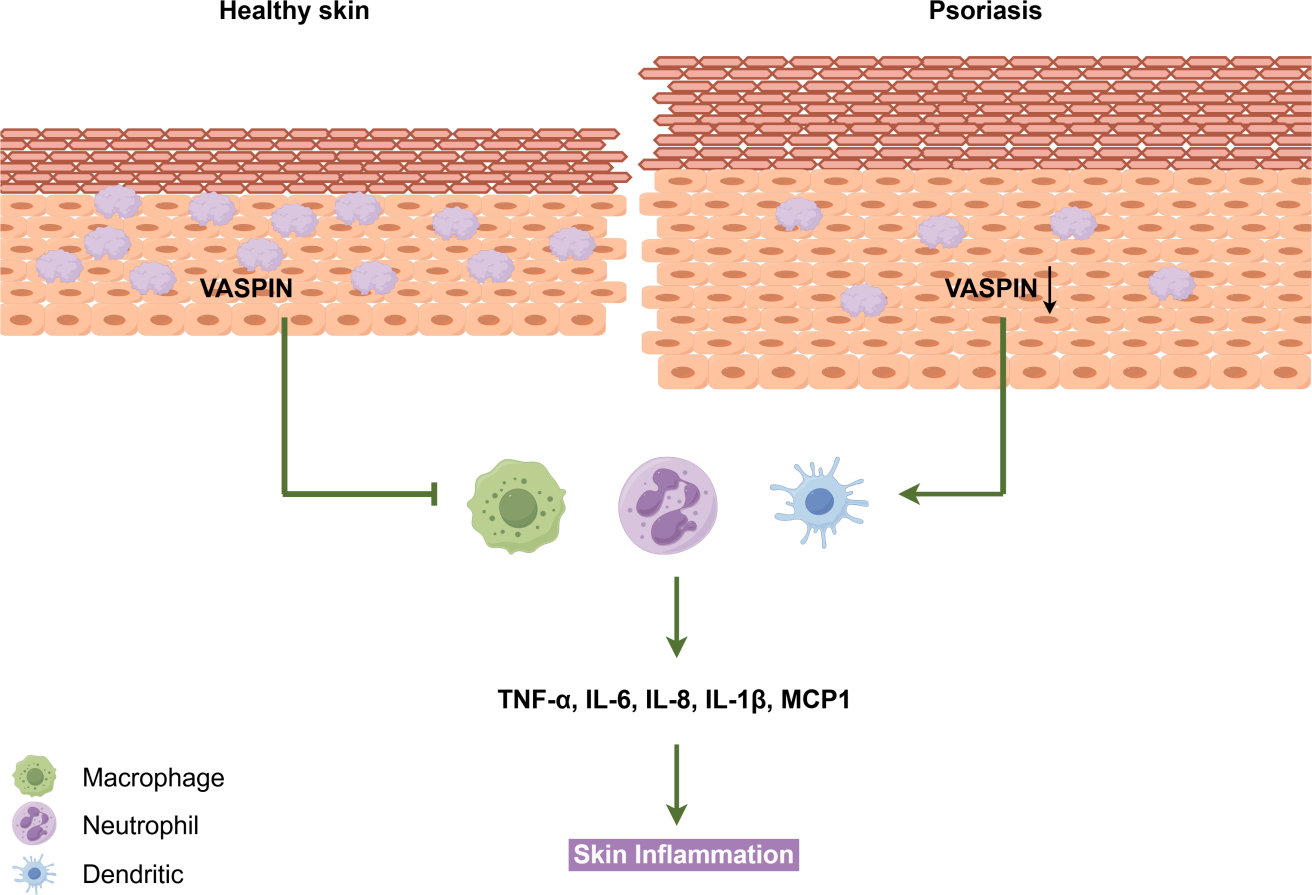
Reduced VASPIN expression and enhanced inflammation in psoriatic skin. Compared with healthy skin, psoriatic lesions exhibit epidermal hyperplasia and decreased VASPIN expression. Reduced VASPIN is associated with increased infiltration and activation of immune cells, including macrophages, neutrophils, and dendritic cells. This leads to elevated production of pro-inflammatory cytokines such as TNF-α, IL-6, IL-8, IL-1β, and MCP-1, thereby promoting and sustaining skin inflammation.

SERPINA12 plays a critical regulatory role in inflammatory skin diseases, particularly psoriasis, where its deficiency exacerbates immune dysregulation and barrier dysfunction. In human psoriasis (**Figure 2**), lesional skin shows a 61.3% reduction in tissue vaspin compared to healthy controls (99.72 pg/mg vs. 257.34 pg/mg, p < 0.001), and this downregulation correlates with disease severity (24). Mechanistically, vaspin deficiency in keratinocytes upregulates psoriasis signature genes (e.g., CCL20, S100A7) and reduces differentiation markers (e.g., involucrin, loricrin) (16). In co-culture experiments, vaspin-depleted keratinocytes stimulate dendritic cells and macrophages to secrete 2–3-fold higher levels of TNF-α, IL-1β, and IL-8, amplifying the inflammatory cascade (16).

Genetic studies further link *SERPINA12* to psoriasis susceptibility. A case-control study of 96 Turkish psoriasis patients found that the rs2236242 TA genotype is associated with a 2.38-fold increased risk of psoriasis (OR = 2.38, 95% CI = 1.25–4.55, p = 0.007) compared to the TT genotype (29). Additionally, serum vaspin levels correlate with metabolic comorbidities common in psoriasis: psoriatic patients have higher vaspin levels than controls (p < 0.05) and vaspin positively correlates with diastolic blood pressure (r = 0.28, p < 0.05) and triglycerides (p = 0.049) (31, 32). These findings position SERPINA12 as a key node connecting skin inflammation, epidermal differentiation, and systemic metabolic dysfunction in psoriasis.

### 3.3 SERPINA12 and palmoplantar keratoderma

The human *SERPINA12* gene is located on chromosome 14q32.13 and belongs to the serine protease inhibitor (serpin) superfamily. It consists of 5 exons that give rise to a conserved serpin structure, including the characteristic reactive center loop essential for its inhibitory activity (33). Alternative splicing of *SERPINA12* has been reported, potentially generating distinct transcript variants with tissue-specific expression patterns. The genomic organization of *SERPINA12* is consistent with other members of the *SERPINA* gene cluster, suggesting shared regulatory mechanisms. Given that mutations affecting gene structure, exon–intron organization, or splicing have been implicated in several inherited keratinization disorders, understanding the genomic architecture of *SERPINA12* provides an important foundation for exploring its potential role in palmoplantar keratoderma (34).

**Figure 3:**
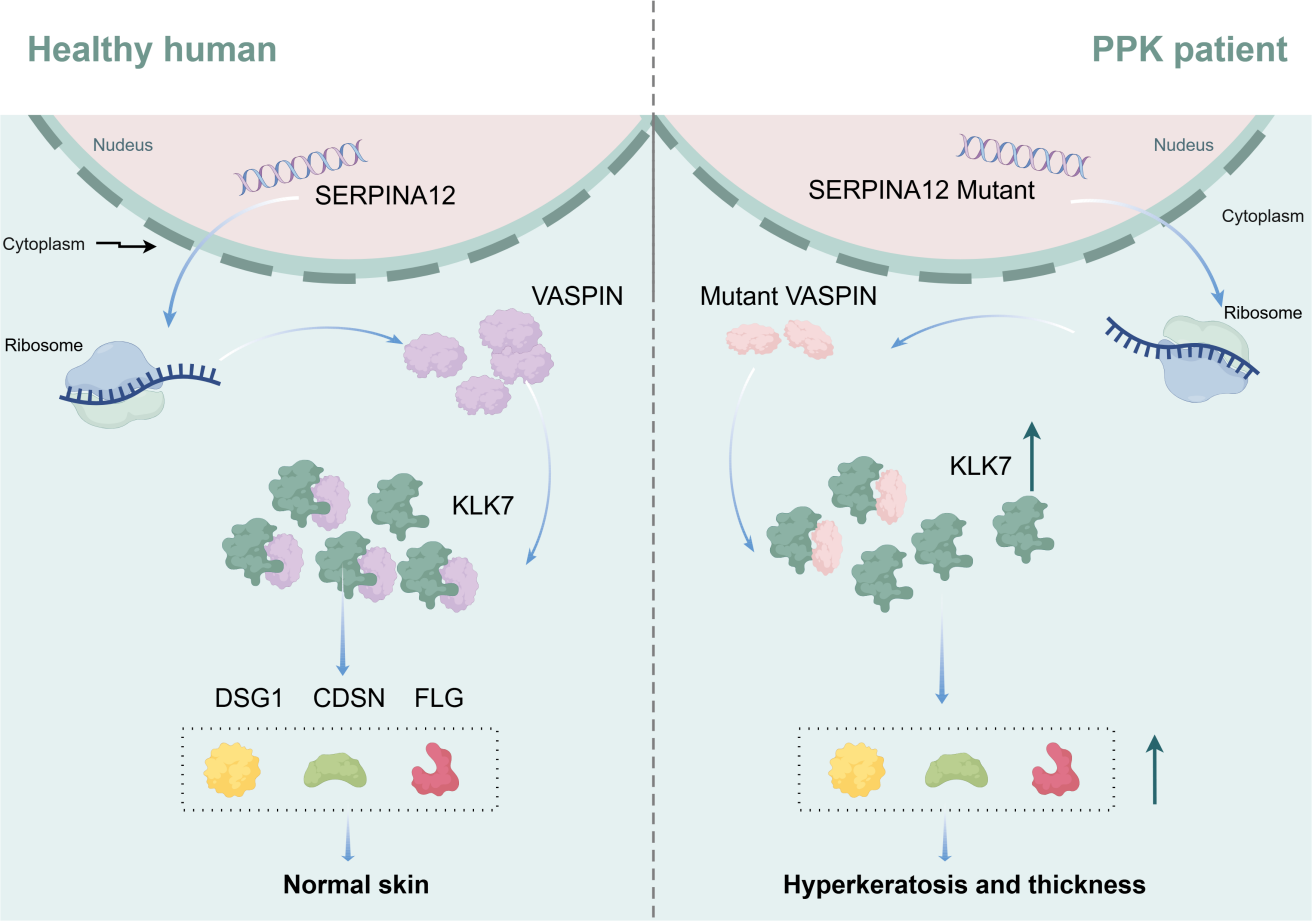
Proposed mechanism of *SERPINA12* mutations in PPK. In healthy individuals, *SERPINA12* encodes VASPIN, which regulates KLK7 activity and maintains the stability of epidermal structural proteins, including DSG1, CDSN, and FLG, thereby preserving normal skin homeostasis. In PPK patients, *SERPINA12* mutations lead to dysfunctional VASPIN, resulting in increased KLK7 activity and excessive degradation or dysregulation of epidermal barrier components. This imbalance contributes to hyperkeratosis and epidermal thickening characteristic of PPK. .

Loss-of-function *SERPINA12* variants are a confirmed genetic cause of autosomal recessive palmoplantar keratoderma (PPK), a group of disorders characterized by thickening of the palms and soles. In 2020, Mohamad et al. first identified homozygous nonsense variants in *SERPINA12* in patients with diffuse, erythematous PPK extending to non-palmoplantar areas (15). These variants reduce SERPINA12 expression in patient skin biopsies, leading to decreased KLK7 inhibition, reduced desmoglein-1 and corneodesmosin levels, and impaired epidermal differentiation (**Figure 3**). In 3D skin equivalents, SERPINA12 downregulation recapitulates the human phenotype, causing marked acanthosis and hyperkeratosis.

**Figure 4:**
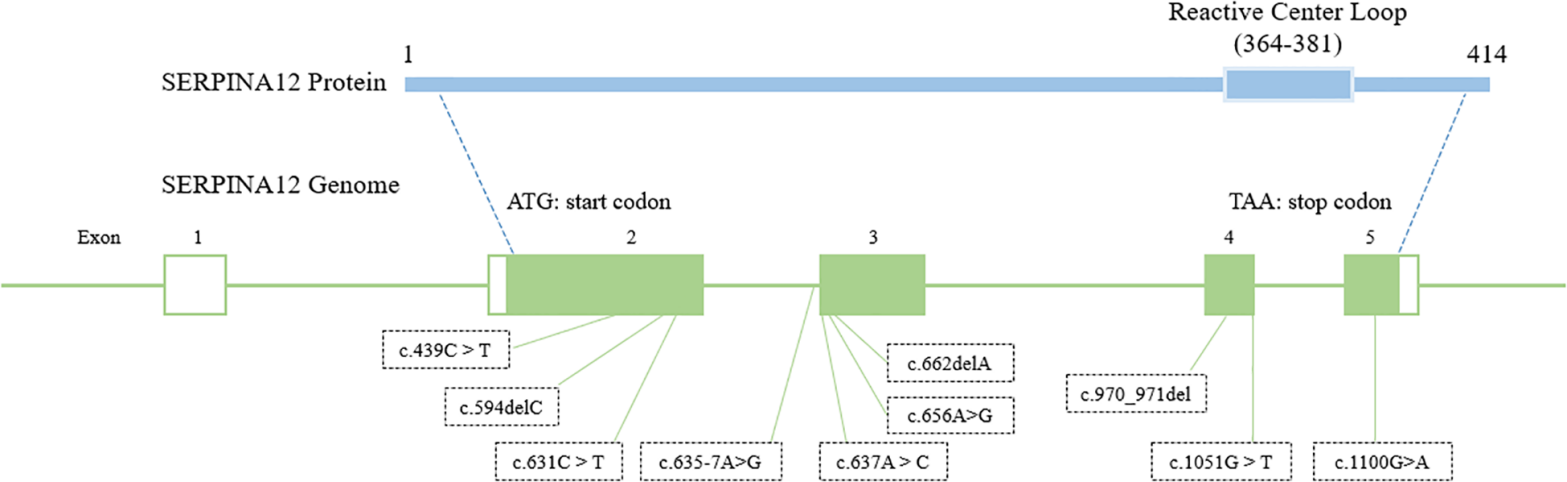
Schematic representation of the *SERPINA12* gene structure and distribution of reported variants. The genomic structure of *SERPINA12* is illustrated with its five exons. All identified variants are mapped to their corresponding exons, highlighting the distribution and localization of mutations across the gene. This diagram summarizes the mutational spectrum of *SERPINA12* reported to date.

Subsequent studies have expanded the genetic and clinical spectrum of *SERPINA12*-related PPK (**Figure 4**; **Table 1**) (34–37). Liu et al. identified four novel variants in *SERPINA12* among six Chinese individuals, thereby broadening the spectrum of *SERPINA12* variants associated with PPK, and demonstrated that c.970_971del is a founder variant (35). In southwestern China, six patients with biallelic *SERPINA12* variants (including the founder variant c.635-7A>G) presented with extensive hyperkeratosis, erythema, and comorbidities like nail dystrophy (38). A Danish cohort identified one family with a *SERPINA12* variant among 76 PPK families, confirming its rarity but clinical significance (39). Notably, *SERPINA12*-related PPK shares phenotypic overlap with Nagashima-type PPK (caused by *SERPINB7* variants), suggesting a common pathway involving serpin-mediated protease inhibition (40). Digenic inheritance of *SERPINB7* and *SERPINA12* variants has also been reported, leading to a severe PPK phenotype (41, 42). These findings establish *SERPINA12* as a critical regulator of palmoplantar epidermal homeostasis.

**Table 1:** Summary of *SERPINA12* mutations associated with hereditary palmoplantar keratoderma.

Year	Patient	*SERPINA12* variant type	Reference
2020	2 from Arab and Indian, respectively	Nonsense: **c.631C > T(p.Arg211Ter); c.1051G > T(p.Glu351Ter);**	Mohamad et al.
2022	6 from China	Nonsense: **c.970_971del(p.Ser324LeufsTer9);^#^** Frameshift: **c.662delA(p.Asn221MetfsTer2);** Missense: **c.656A>G(p.Asp219Gly);** Intronic: **c.635-7A>G;**	Liu et al.
2024	3 from Finland	Nonsense: **c.1100G>A(p.Gly367Glu);** c.631C>T(p.Arg211*);	Brandt et al.
2024	12 from China	Synonymous: **c.637A > C (p.Arg213Arg);** Nonsense: **c.439C > T (p.Gln147Ter);** Missense: **c.364G > A(Benign)** Nonsense: c.970_971del(p.Ser324LeufsTer9); Missense: c.656A>G(p.Asp219Gly);	Liu et al.
2025	1 from Denmark	Frameshift: c.594delC(p.Gly199Alafs*3);	Gram et al.
2025	6 from China	Nonsense: c.970_971del(p.Ser324LeufsTer9); Intronic: c.635-7A>G;^#^	Li et al.
2025	2 from China	Intronic: c.635-7A>G;	Zhang et al.
2025	1 from China	Nonsense: c.970_971del(p.Ser324LeufsTer9);	Huang et al.

Mutations are shown in bold when first reported in the literature. Founder mutations are indicated by a superscript #.

## 4 Diagnostic and therapeutic implications of SERPINA12

### 4.1 Diagnostic biomarkers: SERPINA12 in skin conditions

*SERPINA12* shows promise as a diagnostic biomarker for inflammatory skin diseases and PPK, given its consistent dysregulation in these conditions (**Table 2**). In psoriasis, tissue vaspin levels are 61.3% lower in lesional skin compared to controls (99.72 pg/mg vs. 257.34 pg/mg, p < 0.001), and NB-UVB treatment increases vaspin by 91.4% (p < 0.001) (1, 24). Serum vaspin is also reduced in psoriasis patients (p < 0.05) and correlates with BMI (r = 0.32, p < 0.05) and triglycerides (p = 0.049) (32). For PPK, *SERPINA12* sequencing can confirm a genetic diagnosis: in a Danish cohort, 1/76 families with diffuse PPK had a *SERPINA12* variant (39). Additionally, the rs2236242 TA genotype is associated with a 2.38-fold increased risk of psoriasis (OR = 2.38, 95% CI = 1.25–4.55, p = 0.007), making it a potential risk biomarker (29).

**Table 2:** Cutaneous and serum expression patterns of vaspin (SERPINA12) in dermatological diseases.

Disease	Sample	Expression level	Clinical correlations	Reference
Psoriasis	Patient lesional skin	Decreased relative immunofluorescence intensity in lesional skin compared to non-lesional skin; mRNA expression was reduced to approximately 10% of non-lesional skin;	In the skin of patients with psoriasis, vaspin expression is reduced;	2012. Anja Saalbach
Psoriasis	Patient lesional skin	Relative immunofluorescence intensity in lesional skin was reduced to approximately 40% of that in healthy control skin; mRNA expression was reduced to approximately 10% of healthy control skin;	In the skin of patients with psoriasis, vaspin expression is reduced;	2016. Anja Saalbach
Psoriasis	Patient lesional skin	VASPIN in patient skin (4 mm punch biopsy) measured by ELISA (99.72 pg/mg ± 12.11 pg/mg) was found to be significantly lower compared to after narrowband ultraviolet B radiation (257.34 pg/mg ± 28.11 pg/mg);	Vaspin levels were negatively correlated with PASI scores;	2019. Khadiga Sayed Sayed
Psoriasis	Serum	The serum vaspin levels 35.21 (7.88–411.60) of patients with psoriasis are lower than those of normal individuals 11.53 (0.01–94.14). After treatment, these levels will lightly rise again to 15.08 (0.38–1746.00);	Median vaspin levels decreased with the severity of skin lesions; The vaspin level correlated with BMI of psoriatic patients (p < 0.05), with cholesterol and triglycerides levels (p = 0.054, p = 0.049, respectively);	2022. Paulina Kiluk
Psoriasis	Serum	The serum concentration of soluble vaspin was significantly lower in psoriatic patients compared to healthy controls (1.33 ± 0.32 pg/mL, 1.72 ± 0.39 pg/mL, respectively; P<0.001);	Vaspin may play a role in the pathogenesis of psoriasis and can be used as markers of the disease;	2016. Arzu Ataseven
Systemic sclerosis(SSc)	Serum	SSc patients with digital ulcers were associated with a significant decrease of serum vaspin levels of 0.030 (0.020–0.039) compared to those SSc patient without digital ulcers of 0.049 (0.034–0.088);	Vaspin may be involved in the development of DU in SSc patients;	2015. Shunsuke MIURA
PPK	PPK patient’s skin	Relative immunofluorescence intensity of vaspin in patient’s skin was reduced to approximately 20%-40% compared to normal skin;	Loss-of-function mutations in *SERPINA12* reduce the expression of vaspin in patients’ skin;	2020. Janan Mohamad

While *SERPINA12*’s diagnostic utility is still emerging, its tissue-specific expression and correlation with disease activity make it a strong candidate. For example, in IgA vasculitis, skin transcriptomic analysis identified *SERPINA12* as part of a dysregulated inflammatory signature, though serum levels were not significantly altered (43). Future studies should validate *SERPINA12* as a biomarker in larger cohorts, particularly for early-stage psoriasis and PPK, where diagnosis can be challenging due to phenotypic overlap with other conditions.

### 4.2 Therapeutic targeting of SERPINA12 in skin disease management

SERPINA12’s anti-inflammatory and barrier-protective effects make it a promising therapeutic target for inflammatory skin diseases and PPK. Currently, no specific pharmacological agents directly targeting *SERPINA12* have been approved or entered clinical application. Most available evidence is limited to experimental studies using recombinant vaspin, which has demonstrated protective effects against inflammation, oxidative stress, and metabolic dysfunction in animal models (44, 45). In clinical settings, several commonly used drugs, such as statins, metformin, and insulin sensitizers, have been shown to indirectly modulate circulating vaspin levels, suggesting that vaspin may function as a downstream mediator of metabolic and anti-inflammatory pathways (46).

Emerging therapeutic strategies may include recombinant vaspin supplementation, targeting its receptor LRP1, or modulating downstream signaling pathways such as PI3K-AKT and NF-κB. However, these approaches remain largely theoretical, and further studies are required to evaluate their safety, specificity, and clinical efficacy (45, 47).

In psoriasis, exogenous vaspin application reduced myeloid cell infiltration in a mouse model of psoriasis-like inflammation (16), suggesting that vaspin replacement could alleviate symptoms. Given that NB-UVB upregulates vaspin (24), combining vaspin therapy with phototherapy may enhance efficacy. For PPK, gene replacement therapy could restore *SERPINA12* function: in HCC, intravenous rAAV8-sh*SERPINA12* reduced tumor growth and sensitized cells to sorafenib (26), a strategy that could be adapted for PPK using skin-specific AAV vectors.

SERPINA12’s interaction with heparin also offers therapeutic opportunities. Vaspin binds heparin with high affinity (K_D = 21 ± 2 nM) via basic residues on β-sheet A, and heparin accelerates its inhibition of KLK7 (48). Topical heparin-based formulations could enhance vaspin’s local activity, particularly in psoriasis where the skin barrier is compromised. Additionally, small molecules that mimic vaspin’s protease inhibitory domain or stabilize its structure could be developed to treat *SERPINA12*-deficient PPK. Future preclinical studies should test these approaches in mouse models of psoriasis and PPK to evaluate safety and efficacy.

Given that SERPINA12 regulates multiple signaling pathways, including NF-κB, PI3K-AKT, and MAPK, broad modulation of this molecule may raise concerns regarding off-target or systemic effects. These pathways are involved in diverse physiological processes; therefore, therapeutic strategies targeting SERPINA12 should consider tissue specificity and dosage control to minimize unintended effects (7).

### 4.3 SERPINA12 in personalized medicine for skin disorders

*SERPINA12*’s genetic and functional heterogeneity makes it a candidate for personalized medicine in skin disorders. For psoriasis, the rs2236242 genotype could stratify patients: those with the TA genotype (2.38-fold higher risk) may benefit from vaspin-targeted therapies, while those with the TT genotype may respond better to conventional treatments (29). In PPK, genetic testing for *SERPINA12* variants can guide family planning and early intervention: carriers of pathogenic variants can be counseled on the 25% recurrence risk for autosomal recessive PPK (15).

Additionally, SERPINA12 expression levels could predict treatment response. In psoriasis, patients with higher baseline vaspin levels may respond better to NB-UVB, as the therapy upregulates vaspin (24). For metastatic melanoma, if SERPINA12 is found to drive AKT signaling, patients with high SERPINA12 expression could be treated with AKT inhibitors. Personalized medicine approaches for SERPINA12-related skin diseases will require large-scale genotyping and expression profiling studies to establish robust biomarkers and treatment algorithms.

## 5 Technological advances in SERPINA12 research

### 5.1 High-throughput screening for SERPINA12 in skin

High-throughput screening (HTS) technologies offer the potential to identify small molecules that modulate SERPINA12 function or expression in skin cells. While no HTS studies have focused on SERPINA12 to date, analogous approaches for other serpins provide a blueprint. For example, a HTS of 44,000 compounds identified MCU-i4 and MCU-i11 as inhibitors of mitochondrial calcium uptake (49). A similar screen could target SERPINA12’s protease inhibitory activity or its interaction with GRP78 or LRP1 (44, 47). For instance, compounds that stabilize SERPINA12’s reactive center loop (RCL) could enhance its inhibition of KLK7, while molecules that block SERPINA12-GRP78 binding could suppress AKT signaling in cancer.

HTS can also identify regulators of SERPINA12 expression. In HCC, TCF7L2 binds to the SERPINA12 promoter to drive transcription (26); a screen for TCF7L2 inhibitors could reduce SERPINA12 levels in cancer cells. For psoriasis, where SERPINA12 is downregulated, compounds that activate its promoter (e.g., via PPARγ, which regulates adipokine expression) could restore vaspin levels. Future HTS studies should use keratinocytes or skin organoids as model systems to ensure physiological relevance.

### 5.2 Imaging techniques for SERPINA12 localization in skin

Advanced imaging techniques enable precise localization of SERPINA12 in skin, which is critical for understanding its tissue-specific functions. Immunohistochemistry (IHC) has been used to detect vaspin in the suprabasal epidermis (16), but higher-resolution methods like confocal laser scanning microscopy (CLSM) and mass spectrometry imaging (MSI) offer greater detail. CLSM can visualize vaspin in live skin at cellular resolution: a Mirau-based CLSM device achieves 1.5 μm resolution, allowing visualization of vaspin in keratinocyte layers (50). MSI can map vaspin’s spatial distribution in skin biopsies: a 5 μm resolution MALDI-MSI study localized ceramides and cholesterol in the stratum corneum, a technique that could be adapted for vaspin (51).

*In vivo* imaging techniques like line-field confocal optical coherence tomography (LC-OCT) can also track vaspin dynamics during treatment. LC-OCT achieves 1.5 μm resolution and 17 fps acquisition speed, making it suitable for real-time monitoring of vaspin expression in psoriasis patients undergoing NB-UVB (50). Additionally, magnetic resonance imaging (MRI) with skin markers could visualize vaspin’s distribution in deep skin layers, though this requires labeled vaspin probes. These imaging technologies will advance our understanding of SERPINA12’s localization and function in healthy and diseased skin.

A vaspin-specific cognate aptamer pair (V1 and V49) has been developed using graphene oxide–based SELEX, enabling high-affinity and site-specific dual binding to vaspin. This aptamer system has been applied in sandwich-type surface plasmon resonance (SPR) and quantum dot–based fluorescence imaging assays, allowing sensitive detection of vaspin in both buffer and human serum with a detection down to 3.5 ng/ml in buffer and 4.7 ng/ml in human serum samples, and demonstrating potential utility in early diagnosis of type 2 diabetes (52).

### 5.3 Genetic and proteomic approaches to study SERPINA12 in skin

Genetic and proteomic approaches have been instrumental in unraveling SERPINA12’s role in skin biology. Whole-exome sequencing (WES) identified the first pathogenic *SERPINA12* variants in PPK patients (15), and subsequent WES studies expanded the list of variants to include missense, nonsense, and splice-site mutations (38). Sanger sequencing has been used to validate these variants in family members: in a Turkish cohort, 96 psoriasis patients were genotyped for rs2236242, revealing a 2.38-fold increased risk for the TA genotype (29).

Proteomic studies have characterized SERPINA12’s interactions with target proteases and signaling molecules. For example, co-immunoprecipitation (co-IP) showed that vaspin binds KLK7 and KLK14 (20, 21), while microscale thermophoresis (MST) measured its high-affinity binding to heparin (K_D = 21 ± 2 nM) (48). Mass spectrometry (MS) identified the reactive center loop cleavage site of vaspin (between M378 and E379), critical for its inhibitory activity (33). Future studies should combine single-cell RNA sequencing (scRNA-seq) with proteomics to map SERPINA12 expression in specific skin cell types and identify cell-specific interactions.

## 6 Controversies and challenges in SERPINA12 research

### 6.1 Debates on the role of SERPINA12 in skin physiology

Despite growing evidence for SERPINA12’s role in skin biology, key debates remain. One controversy is whether SERPINA12 acts primarily as a protease inhibitor or an adipokine in the skin. While its inhibition of KLK7 and KLK14 is well-documented (20, 21), its adipokine functions—such as regulating insulin sensitivity—are less clear in skin cells. For example, vaspin improves glucose metabolism in adipose tissue (21), but its effect on keratinocyte glucose uptake is unknown. Another debate is the direction of SERPINA12’s regulation in psoriasis: while most studies report downregulation (16, 24), one study found higher serum vaspin levels in psoriasis patients compared to controls (31). This discrepancy may reflect differences in patient obesity status (since vaspin is adipocyte-derived) or sample type (tissue vs. serum).

Additionally, the functional overlap between SERPINA12 and other serpins (e.g., SERPINB7) in PPK is debated. While both serpins inhibit KLK7, *SERPINB7* variants cause Nagashima-type PPK, which is phenotypically distinct from *SERPINA12*-related PPK (e.g., less erythema) (40). Whether these differences stem from tissue-specific expression or distinct protease specificities requires further investigation. Resolving these debates will require standardized experimental protocols, including consistent sample collection (lesional vs. non-lesional skin) and obesity matching in patient cohorts.

### 6.2 Challenges in targeting SERPINA12 for skin therapies

Targeting SERPINA12 for skin therapies faces several challenges. First, its dual role as a protease inhibitor and signaling molecule raises the risk of off-target effects. For example, vaspin’s inhibition of KLK7 is critical for barrier function, but excessive inhibition could lead to impaired desquamation and hyperkeratosis. Second, delivering SERPINA12 to the skin is challenging due to the stratum corneum barrier. While topical formulations are ideal, large proteins like vaspin (≈47 kDa) have poor skin penetration. Nanoparticle-based delivery systems—such as polymeric nanoparticles or liposomes—could enhance penetration, but their safety and efficacy in skin need validation (53).

Third, *SERPINA12*’s genetic heterogeneity complicates therapy development. For PPK, different pathogenic variants (nonsense, splice-site) may require different approaches: gene replacement for loss-of-function variants vs. exon skipping for splice-site variants. Fourth, the lack of animal models for *SERPINA12*-related skin diseases hinders preclinical testing. While mouse models of psoriasis exist (16), no mouse model of *SERPINA12*-deficient PPK has been reported. Developing such models will be critical for testing therapeutic strategies.

### 6.3 Ethical considerations in *SERPINA12* genetic research

Genetic research on *SERPINA12* raises ethical considerations related to informed consent, data sharing, and incidental findings. For PPK, genetic testing can reveal carrier status in family members, which may have psychological implications (e.g., anxiety about passing the variant to children). Informed consent processes must clearly disclose the risks and benefits of testing, including the possibility of incidental findings (e.g., variants in other disease genes) (54). Additionally, data sharing from SERPINA12 studies must protect patient privacy, particularly since genetic data is personally identifiable. Using anonymized datasets and secure cloud storage can mitigate this risk (55).

Another ethical consideration is the equitable access to SERPINA12-based therapies. Since PPK is a rare disease, therapies may be expensive, limiting access for low-income patients. Orphan drug designations and government subsidies can help address this, but global collaboration is needed to ensure affordability (56). Finally, genetic research on *SERPINA12* in underrepresented populations (e.g., southwestern China) must involve community engagement to avoid exploitation. Researchers should work with local stakeholders to design studies that address community needs and share benefits (e.g., capacity building, free testing) (57).

## 7 Future directions in SERPINA12 skin research

### 7.1 Emerging trends in SERPINA12 functional studies

Emerging trends in SERPINA12 research focus on its cell-specific functions, signaling pathways, and interactions with other molecules. Single-cell RNA sequencing (scRNA-seq) will map SERPINA12 expression in keratinocytes, fibroblasts, and immune cells, revealing cell-specific roles. For example, scRNA-seq could determine whether vaspin is expressed in epidermal stem cells and its effect on stem cell proliferation. Another trend is the investigation of SERPINA12’s non-canonical functions, such as its interaction with GRP78 in cancer cells (26). Studying this interaction in skin cancer could identify novel therapeutic targets.

Additionally, structural biology studies will elucidate SERPINA12’s mechanism of action. The crystal structure of cleaved vaspin has been solved (33), but the structure of the uncleaved form (which is active) remains unknown. Solving this structure will reveal how vaspin interacts with its target proteases and heparin. Finally, epigenetic regulation of SERPINA12—such as DNA methylation or histone modifications—will be explored, as epigenetic changes are common in inflammatory skin diseases. For example, hypomethylation of the SERPINA12 promoter could explain its upregulation in obesity-related psoriasis.

### 7.2 Potential for SERPINA12 in regenerative skin medicine

SERPINA12 has potential applications in regenerative skin medicine, particularly for wound healing and barrier repair. Its anti-inflammatory activity and regulation of epidermal differentiation make it ideal for treating chronic wounds, which are characterized by persistent inflammation and impaired re-epithelialization. For example, topical vaspin application could reduce inflammation in diabetic foot ulcers and promote keratinocyte migration. Additionally, SERPINA12 could be incorporated into skin substitutes (e.g., 3D bioprinted skin) to enhance barrier function: in 3D skin equivalents, SERPINA12 downregulation causes hyperkeratosis (15), so overexpression could improve differentiation.

Furthermore, SERPINA12’s interaction with heparin could be exploited to enhance skin regeneration. Heparin-based scaffolds are used in wound healing to promote angiogenesis and cell migration (48); combining these scaffolds with vaspin could provide both structural support and protease inhibition. Future studies should test vaspin-containing skin substitutes in animal models of chronic wounds to evaluate their regenerative potential.

### 7.3 Future prospects for SERPINA12 in skin health and disease

The future of SERPINA12 research in skin health and disease is promising, with several key prospects. First, clinical trials will evaluate vaspin-targeted therapies for psoriasis and PPK. For example, a phase I trial could test the safety of topical vaspin in psoriasis patients, while a phase II trial could assess its efficacy in reducing PASI scores. Second, SERPINA12 will be integrated into multi-biomarker panels for skin diseases. For example, combining vaspin with other inflammatory markers (e.g., IL-6, TNF-α) could improve diagnostic accuracy for psoriasis. Third, SERPINA12’s role in skin aging will be clarified, potentially leading to anti-aging therapies that target vaspin to maintain barrier function and reduce inflammation.

Finally, global collaborations will expand our understanding of *SERPINA12*’s genetic and phenotypic diversity. For example, the International PPK Registry could collect data on *SERPINA12* variants from around the world, revealing population-specific founder effects (e.g., c.635-7A>G in southwestern China) (58). These collaborations will accelerate the translation of SERPINA12 research into clinical practice, improving outcomes for patients with skin disorders.

In conclusion, SERPINA12 is a multifunctional protein with critical roles in skin biology and disease. Its regulation of protease activity, inflammation, and epidermal differentiation makes it a promising target for diagnostics and therapies. While challenges remain—including debates about its function and delivery hurdles—ongoing research will unlock its full potential in personalized medicine and regenerative skin care.
